# Beginning EFL Teachers' Emotional Labor Strategies in the Chinese Context

**DOI:** 10.3389/fpsyg.2021.737746

**Published:** 2021-08-17

**Authors:** Hanxi Li, Honggang Liu

**Affiliations:** School of Foreign Languages, Northeast Normal University, Changchun, China

**Keywords:** emotional labor strategy, beginning EFL teacher, Mainland China, structure validation, levels of emotional labor strategies

## Abstract

Teachers' emotional labor strategies have been explored in different cultural contexts. However, beginning English as a foreign language (EFL) teachers have received scant research attention. This study seeks to conceptualize emotional labor strategies among Chinese beginning EFL teachers and investigate their general profiles. The developed Beginning EFL Teachers' Emotional Labor Strategy Scale was assessed among 484 Chinese beginning secondary school EFL teachers. A final 20-item scale was obtained after a series of reliability (e.g., item analysis, internal consistency, composite reliability) and validity tests (e.g., construct validity, convergent validity, discriminant validity). The confirmatory factor analysis results supported the four-dimensional structure of emotional labor strategies in the beginning EFL teacher sample, encompassing surface acting, deep acting, positive consonance, and negative consonance. In addition, participants reported high levels of positive consonance and deep acting, and low levels of surface acting and negative consonance. Possible reasons for this are discussed in reference to the EFL educational context in China. The findings provide insights into sustainable development for Chinese beginning secondary school EFL teachers.

## Introduction

Since the mid-1990's, educational researchers have paid increasing attention to the important role of teachers' emotions in their teaching. Teachers are emotionally engaged in their work, and teaching practices carry emotions (Zembylas, 2006). Recent studies have indicated that teachers' emotional behavior, such as stroke and emotioncy, influences students' communicative competence (Pishghadam and Ebrahimi, 2020), learning motivation (Pishghadam et al., 2021a), willingness to attend classes (Pishghadam et al., 2021b), and emotions experienced in classes (Derakhshan et al., 2021). Among the topics related to teacher emotion studies (Wang et al., 2021) is emotional labor. The sociologist Hochschild (1983) originally conceptualized this term as “the management of feeling to create a publicly observable facial and bodily display” (p. 7).

Educational researchers have extensively investigated the characteristics of teachers' emotional labor in the school context. So far, they have explored the nature, dimensions, antecedents, moderators, and outcomes of teachers' emotional labor (Zembylas, 2006; Benesch, 2017; Wang et al., 2019). Although these studies have been conducive to the theoretical development of teachers' emotional labor, the dimensions of teachers' emotional labor, especially their emotional labor strategies, require more precise definitions (Yin, 2016; Wang et al., 2019). Moreover, researchers (e.g., Cukur, 2009; Yin, 2012; Wang et al., 2019) have called for more quantitative research to define and measure teachers' emotional labor or emotional labor strategies. Beginning EFL teachers' emotional labor strategies in the Chinese context appear to be one.

Comparatively speaking, the progress of research into English as a foreign language (EFL) teachers' emotional labor has been relatively slow. Emotional labor was not introduced into EFL studies until the early 21st century (Gkonou and Miller, 2017). Further, most studies on EFL teachers' emotional labor have used qualitative research methods (e.g., Loh and Liew, 2016; Yin, 2016; Benesch, 2017). While they have provided rich descriptions of the emotional labor processes of experienced EFL teachers, they have largely overlooked beginning EFL teachers (Kocabaş-Gedik and Hart, 2020). There is a consensus that beginning teachers will encounter various problems in the early stage of their teaching career (Jiang et al., 2020). For example, they must negotiate their teacher identity. While familiarizing themselves with teaching materials and how to manage the classroom, they should consider how to build harmonious relationships with others, including students, colleagues, administrators, and parents (Hargreaves, 2005; Kocabaş-Gedik and Hart, 2020). They experience a whirlwind of emotions, from initial tension and excitement to helplessness, worry, anxiety, distress, vulnerability, and even anger (Xu, 2013). Some beginning teachers become frustrated, lose confidence in the teaching profession, and choose to leave the profession. In fact, attrition rates reach their peak in their first 10 years of a teaching career (Macdonald, 1999). Therefore, this early phase plays a vital role in decreasing attrition and promoting teachers' professional development.

Against this backdrop, the present study constitutes an attempt to conceptualize Chinese beginning secondary school EFL teachers' emotional labor strategies using a quantitative research method. Our rationale for choosing this topic is as follows. Controversy regarding dimensions of emotional labor strategies exists. Moreover, due to the lack of quantitative measures for EFL teachers' emotional labor strategies, Chinese beginning EFL teachers' usage of emotional labor strategies is still in unknown state. Hence, by developing the Beginning EFL Teachers' Emotional Labor Strategy Scale and combining it with exploratory factor analysis (EFA) and confirmatory factor analysis (CFA), we aim to clarify the construct of beginning EFL teacher's emotional labor strategy and redefine its nature. Hopefully, the findings will help fill research gaps and enable us to learn the general files of Chinese beginning EFL teachers' usage of emotional labor strategies. The remainder of the article is organized as follows. Section Literature Review introduces the background for the present research by overviewing relevant research and key theoretical concepts along with a statement of the research questions at the end. Section Method presents research methodology of the study. Section Results reports the results, while Section Discussion discusses the findings. Section Conclusion and Implications concludes the article by summarizing the main findings and providing the implications for future research.

## Literature Review

According to the research purposes stated above, literature review will provide theoretical support for this study. This section mainly introduces the key concepts guiding this research, including emotional labor and emotional labor strategies. It also presents the controversy within emotional labor strategy research and discusses the progress of EFL emotional labor studies.

### Defining EFL Teacher's Emotional Labor Strategies

Yin et al. (2017) defined the emotional labor of teaching as “the effort, planning, and control needed for teachers to express organizationally desired emotion during their interpersonal transactions with students and others in classroom and school settings” (pp. 127–128). This definition implicitly reflects that interaction will create emotional labor for teachers and that there exist organizational expectations for teachers' emotional behavior. Benesch (2017) laid out the conceptualization of emotional labor in the field of English language teaching and defined emotional labor as “English language teachers' self-monitoring to achieve appropriate emotions guided by institutional policies and professional guidelines”; besides, they may “resist or comply with those rules” when responding to challenging pedagogical situations (p. 53). This definition takes into account the realities of language teaching. From the above two definitions, we can see that emotional labor is closely connected with teaching and interaction, controlled by social and cultural expectations. The emotional rules are not fixed, implying that teachers could choose to breach them.

Emotional labor strategies are methods or skills that individuals employ to manage their emotions (Yin, 2016). Previous empirical studies (Li et al., 2012; Yin, 2012) verified that teachers engage in emotional labor by utilizing surface acting, deep acting, and expression of felt emotion. Surface acting refers to a strategy whereby teachers “stimulate emotions that are not actually felt or change the outward expression of felt emotion” (Cukur, 2009, p. 561). Deep acting means “the emotion management process in which teachers try to modify their felt emotions using some cognitive techniques (e.g., distraction and self-persuasion)” to display appropriate emotions and behavior (Yin, 2012, p. 452). Both surface acting and deep acting manifest teachers' efforts to be consistent with emotional rules and the gap between felt and displayed emotions. In contrast, the third strategy, expression of felt emotion, is when felt emotions are in line with emotional rules.

Some researchers (e.g., Mikolajczak et al., 2007; Cukur, 2009; Yang et al., 2019) have argued that in nature, emotional labor strategies are methods for managing emotion, and previous studies failed to consider strategies in which individuals ignore emotional rules on purpose to manage their emotions. That is, researchers have expressed doubts about the dimensions of emotional labor strategies. According to Mikolajczak et al. (2007), an emotional labor strategy is a four-dimensional construct composed of surface acting, deep acting, positive consonance, and negative consonance. The connotation of positive consonance is basically consistent with the expression of felt emotion, whereas negative consonance refers to a situation where felt and expressed emotions concord but are inconsistent with emotional rules. Likewise, Cukur (2009) categorized four dimensions of emotional labor strategies: surface acting, deep acting, automatic emotion regulation (i.e., expression of felt emotion), and emotional deviance. He developed a 20-item Teacher Emotional Labor Scale and distributed it to 190 high school teachers in Turkey. CFA results supported the four-dimensional structure. In this respect, Mikolajczak et al. (2007) and Cukur (2009) added a new strategy in which individuals manage emotions by violating emotional rules, thus “reflecting divergent consequences for different types of emotional labor” (Cukur, 2009, p. 562). Yang et al. (2019) developed a Chinese version of the emotional labor strategy scale and verified the four-dimensional construct of surface acting, deep acting, expression of naturally felt emotions, and emotion termination. Emotion termination means that individuals “take efforts to stop conveying their internal and external emotions” when they encounter conflict situations (Yang et al., 2019, p. 6). By resorting to this strategy, individuals may achieve organizational goals while maintaining harmony to a large extent.

The ongoing debate over the division of emotional labor strategies has disclosed that they have different structures in different cultural contexts and, therefore, have various connotations. The controversy over whether there are three or four dimensions lies in whether the focus is on the outcomes accordant to emotional rules or the ways of managing emotions. In particular, researchers in favor of three-dimensional strategies emphasize the outcomes of emotional labor and consider emotional rules as standard. On the contrary, researchers who support the four-dimensional structure believe that it should cover the violation of emotional rules, conforming to the regulation process.

### Empirical Studies on EFL Teachers' Emotional Labor Strategies

Up to now, empirical studies on EFL teachers' use of emotional labor strategies have analyzed the teachers' motivations for applying these strategies and the processes and outcomes of applying them. Adopting a dialogical perspective and drawing on positioning theory, Gkonou and Miller (2017) investigated why and how eight EFL teachers in Greece addressed students' language anxiety. After analyzing their semi-structured in-depth interview data, they found that an ethic of care motivated teachers to emotionally attend to students. Teachers alleviated students' language anxiety by establishing a positive relationship with the students, but when the teachers used various strategies to alleviate the students' anxiety and cared for the students according to the requirements of emotional rules, it usually meant that the teachers had to perform continuous emotional labor. King (2016) conducted semi-structured interviews with five experienced Japanese private university EFL teachers to assess their use of surface acting, deep acting, and suppression of negative emotions. His research led to several conclusions. For example, it is necessary to create a positive and caring teacher–student relationship, and teachers' emotions toward students will affect their choice of teaching strategies. To create a positive learning atmosphere in class, teachers usually suppress negative emotions, such as anger, frustration, and irritation. By engaging in emotional labor, such as showing exaggerated positive emotions, teachers try to stimulate students' learning motivation. Teachers will use emotional labor strategies to manage their emotions and to achieve educational goals. Zhang and Zhu (2008) examined Chinese college instructors' use of emotional labor strategies and their effects on teacher satisfaction and burnout. They distributed a questionnaire that 164 EFL instructors filled out. The quantitative results showed that surface acting had deleterious effects on teachers' burnout and teaching satisfaction, while deep acting and expression of naturally felt emotions had positive effects. These results support that the use of emotional labor strategies can be both positive and negative for instructors, depending on which strategy is more prominent. The above empirical studies confirmed the important role of emotional labor strategies played in EFL teaching process (Greenier et al., 2021).

The few studies that have focused on beginning EFL teachers have preliminarily analyzed the emotional dilemmas they encounter and the strategies they utilize to manage emotions. However, there is no fixed conclusion about the specific dimensions of their strategies. Xu (2013) conducted a narrative case study on three beginning EFL teachers at a secondary school in China. Based on qualitative data collected through semi-structured interviews and teachers' reflective journals, the study depicted the teachers' emotional experiences with students, colleagues, parents, and leaders in the 1st year of teaching. The interaction with students was the source of their positive emotions, but teachers also lost their temper over students' mistakes. The emotional experiences of communicating with colleagues were relatively complex, which was reflected in respect for mentors, concern about colleagues' jealousy, and so on. Some teachers felt the joy of being admired by parents, while some felt indignation owing to parents' unreasonable demands. Finally, they experienced the interaction with administrators as encouraging and inspiring.

Tejeda et al. (2016) pointed out that EFL teaching is full of emotional labor, and their research confirmed that beginning EFL teachers reduce the influence of negative emotions through emotion regulation. Drawing on data from semi-structured interviews and observations, they summarized the strategies five EFL teachers in Mexico used for emotion regulation, including selecting situations, cognitive change, modifying the emotional experience and expression. Adopting a poststructuralist approach, Kocabaş-Gedik and Hart (2020) did a longitudinal case study to explore two beginning EFL teachers' emotional labor experiences. They collected data over 6 months and focused on their 1st year at a university in Turkey. Journal entries, interviews, and field notes revealed that some individual factors led to different emotional states and emotional labor experiences—namely, emotional freedom and emotional suffering. Influencing factors included educational background, support from colleagues and the institution, local language ability, professional expectations, and career planning. Teachers who have teaching experience and knowledge of teaching methods can better devote themselves to the teaching practice and regulate their emotions to adjust their emotional distress. Furthermore, teachers who have a better command of the local language and are supported in their work feel less emotional distress and enjoy more emotional freedom.

Most extant studies on EFL teachers' emotional labor strategies have focused on experienced teachers or a whole group of teachers and have paid less attention to beginning EFL teachers. Thus far, the research has mainly explored the reasons why EFL teachers engage in emotional labor, the types of emotional labor strategies they use, the deep connection with teaching, and the influence of emotional labor strategies. Compared with experienced or expert EFL teachers, beginning teachers face more challenges and difficulties in their daily work due to lack of experience and help, and they may experience more negative emotions. The beginning teachers in extant studies tended to come from different cultural backgrounds, such as China, Mexico, and Turkey. Consequently, cultural differences and different teaching targets caused them to encounter varying situations of emotional labor, and their emotional labor exhibited unique trajectories.

Considering the professional development and turnover intention of beginning teachers, more attention should be paid to their emotional labor status to help them improve their emotion regulation ability. In reviewing previous studies, it became apparent that most of the empirical literature on beginning EFL teachers has focused on their emotional experiences and challenges and has largely overlooked their use of emotional labor strategies. Specifically, it is not clear whether beginning EFL teachers use distinct emotional labor strategies and, thus, what the construct structure of these strategies is. Therefore, more systematic and quantitative studies are required to measure beginning EFL teachers' emotional labor strategies.

In addition, compared with studies in other cultural backgrounds, the study of Chinese beginning EFL teachers' emotional labor strategies has proceeded slowly. The international literature contains little research on the measurement of emotional labor strategies for Chinese beginning EFL teachers. Therefore, this study is intended to provide a specific conceptualization of emotional labor strategies, clarifying the construct that Chinese beginning EFL teachers in secondary schools use, based on the factorial validity results acquired from CFA. The study also aims to describe the status of emotional labor strategies in use.

Correspondingly, the following two research questions were formulated to guide the current research:

1. What is the structure of the Chinese beginning secondary school EFL teachers' emotional labor strategy?
2. What are the overall and dimensional profiles of their emotional labor strategies?

## Method

### Participants

Beginning teachers are qualified teachers who have <7 years of experience (Lassila et al., 2021). Day (2017) conducted a 4-year investigation of 300 primary and secondary school teachers in Britain. The findings suggested that teachers with 0–3 years of teaching experience need to build a sense of self-efficacy in the classroom. Moreover, teachers may encounter more difficult professional challenges and take on heavier workloads and responsibilities in their 4th to 7th years, leading to decreased confidence in their teaching abilities. Therefore, teachers with <7 years of teaching experience are beginning teachers at an early stage of professional development (Day and Gu, 2010).

Using convenience sampling, 484 beginning EFL teachers from Chinese secondary schools were invited to participate in the study. They were from four municipalities, three autonomous regions (Inner Mongolia Autonomous Region, Guangxi Zhuang Autonomous Region, and Xinjiang Uygur Autonomous Region), and 24 provinces in China. In the sample, there were 39 male (8.1%) and 445 female (91.9%) teachers, and 259 teachers were from junior high schools (53.5%), while 225 teachers were from senior high schools (46.5%). The mean teaching tenure was 2.4 years (*SD* = 1.072).

### Instruments

In this study, a self-developed Beginning EFL Teachers' Emotional Labor Strategy Scale was used to conduct an online survey. A Chinese-language version of the scale was produced to assess beginning secondary school EFL teachers' four types of emotional labor strategies: surface acting (5 items), deep acting (6 items), negative consonance (6 items), and positive consonance (3 items). The scale included three parts: introduction (the purpose and significance of the survey, instructions, and confidentiality commitments), respondents' demographic variables (gender, education level, school type, teaching age), and the main body of the scale (see Appendix A for details). The main body of the scale contained 20 items: 10 items from the general body of emotional labor literature and qualitative research findings and 10 items from existing emotional labor strategy measurements. These items were revised slightly to fit the character of EFL beginning teachers' education setting. For example, the original item of Yang et al.'s (2019) scale, “When there is disagreement with the customer, I will serve according to the customer's requirements without any emotional change,” was changed to “When there is disagreement with the leader, I will choose to follow the leader's requirements without any emotional change” (item 16). The items were rated on a 5-point Likert scale ranging from 1 (never) to 5 (always). The specific information about each dimension's distribution is detailed in Table 1.

**Table 1:** Dimensions, distributions, and examples of the Beginning EFL Teachers' Emotional Labor Strategy Scale.

**Scales**	**Items**	**Item sample**
Surface acting	1–5	Although I was nervous when I encountered English words I didn't know in class, I still tried my best to keep calm (Q01).
Deep acting	6–11	In the first open class, I tried to concentrate on the class, which helped me feel less nervous (Q05).
Negative consonance	12–17	I tend to show my disappointment when students don't master the English grammar points that have been taught many times (Q18).
Positive consonance	18–20	I can open my heart to communicate with students and establish a harmonious relationship with them (Q24).

### Research Procedures and Data Analysis

The first step was to generate items reflecting beginning EFL teachers' emotional labor strategies. To this end, we invited 13 EFL beginning teachers from Chinese secondary schools to participate in in-depth semi-structured interviews. Participants were provided with an electronic information sheet that listed the purpose of this research and explained their rights of confidentiality and withdrawal from the study at any time. The first researcher interviewed each participant by asking open-ended questions: What are the most positive and negative experiences in your interactions with others (students/parents/leaders/colleagues/masters)? How do you regulate these feelings? Subsequently, the interview recordings were transcribed to yield text data, and NVivo 12 was used to identify types of emotional labor strategies (see Appendix B for details). Previous conceptualizations of emotional labor strategies, empirical findings, and existing emotional labor strategy scales (Mikolajczak et al., 2007; Cukur, 2009; Yin, 2016; Benesch, 2017; Yin et al., 2017; Yang et al., 2019) served as the basis for generating a pool of items. Relevant measurement tools included F-QUEL (Mikolajczak et al., 2007), Teacher Emotional Labor Scale (Cukur, 2009), Teacher Emotional Labor Strategy Scale (Yin, 2012), Emotional Labor Scales for Elementary School Teachers (Li et al., 2012), and Chinese Emotional Labor (Yang et al., 2019). An initial scale consisting of 30 items was formed. The overall plan was to construct the scale so that each item would reflect one construct and would be close to the status of beginning EFL teachers. The wording needed to be fairly brief and relatively neutral to make the items easy to understand and to avoid ambiguity. This initial pool of 30 items was revised according to feedback from 20 EFL teachers and three Chinese teachers in secondary schools. We deleted items with ambiguous meanings and modified the expression of some items according to the teachers' suggestions, resulting in a scale of 26 items (see Appendix C for details).

The second step was quantitative data collection, which lasted from November 10 to 21, 2020. The 26-item scale was circulated to beginning EFL teachers via an online survey website called Wenjuanxing. Participants' individual consent was obtained at the start of the survey, and 484 valid scales were collected in total. The third step was to examine the factor structure and report the emotional labor strategy status. In terms of the first research question, series of exploratory and confirmatory factor analyses were conducted using SPSS 26.0 and AMOS 26.0, respectively, to refine and confirm the factor structure. In addition, item analysis, reliability tests (e.g., internal consistency, composite reliability), and validity tests (e.g., convergent validity, discriminant validity) were performed to confirm the psychometric properties of this scale. After a series of adjustments, six items (items 6, 9, 12, 13, 25, and 26) were deleted. With regard to the second research question, descriptive analyses were performed with SPSS 26.0 to describe the level of each emotional labor strategy. The final 20-item scale was used to analyze the emotional labor strategy level of the beginning EFL teachers. Since EFA requires a sample size of at least five times the number of items (Pallant, 2013), we used SPSS 26.0 to randomly select 30% (*N* = 159) of the total sample size—that is, 484 teachers. Both item analysis and EFA were performed using the data from this sample. The data of the remaining 325 teachers were used for CFA, reliability tests, validity tests, and descriptive analyses.

Item analysis was first performed to detect whether the items can effectively distinguish participants with different characteristics—that is, the discriminant validity of each item (Wu, 2010a). First, rankings based on participants' total scores in the 26 items were created, and the upper 27% and lower 27% groups were established. Then an independent samples *t*-test was conducted between the high- and low-score groups to compare their responses to each item. The results (see Appendix D) showed that the critical ratio (CR) reached a significant level (*p* < 0.01), indicating that the items were appropriate for further analysis (Green and Salkind, 2014). An item-total correlation analysis was then performed to investigate the correlations between each item and the global scale. The results (also see Appendix D) indicated that the correlation coefficients all reached the benchmark *r* = 0.30 (Pallant, 2013), all reaching a significant level (*p* < 0.01). From this, we can see that the 26-item scale can identify different participants, making it unnecessary to eliminate items.

## Results

### Structure of Beginning EFL Teachers' Emotional Labor Strategies

In this study, both EFA and CFA were used to examine the factor structure of EFL teachers' emotional labor strategies. Before carrying out the analyses, we conducted a test of univariate normality to determine if the data were normally distributed, especially checking skewness and kurtosis for each item. A value between +2 and −2 would indicate normal distribution (Bryman and Cramer, 2001). Skewness and kurtosis values of the 26 items (*N* = 159) were between +2 and −2 (see Appendix E), indicating that the data met the requirements of normal distribution. The Kaiser-Meyer-Olkin (KMO) measure and Bartlett's test of sphericity were conducted to ensure that the data had sufficient correlations to perform EFA. The KMO index was 0.863 and Bartlett's test of sphericity (χ^2^ = 2274.146, df = 253) was significant at a level of 0.000. As the KMO index was above 0.8 and the statistic of spherical test reached a significant level (*p* < 0.001), this justified that the correlation matrix was not a one-dimensional matrix and that the data were suitable for factor analyses.

EFA was conducted to identify the factor structure of emotional labor strategies. The extraction method of principal axis analysis was applied to find latent variables, and the threshold value of the factor loading was set at 0.40 (Green and Salkind, 2014). After four rounds of EFA, three items (items 9, 12, and 13) were removed because their factor loadings were either below 0.40 or their commonality was below 0.30. Thus, a four-factor structure with 23 items was obtained. As Table 2 shows, the eigenvalues of all factors were >1.00, accounting for a total of 58.25% of the variance. If the cumulative percentage of total modeled variance is more than 55%, it means that the extracted factors have a better explained ratio (Plonsky and Gonulal, 2015). The commonality of each item was above 0.230 and the reliability of each factor ranged from 0.839 to 0.926, indicating the scale's high reliability (see Appendix F).

**Table 2:** Results of EFA for emotional labor strategies.

	**Factor 1 positive consonance**	**Factor 2 negative consonance**	**Factor 3 surface acting**	**Factor 4 deep acting**	**Commonality**
Q25 Students' positive response made me enthusiastic.	**0.902**	0.055	0.001	−0.012	0.80
Q26 Students' improved levels made me confident.	**0.879**	0.048	−0.020	−0.003	0.78
Q22 I love my students genuinely.	**0.863**	0.029	0.053	0.046	0.77
Q24 I openly communicated with students.	**0.806**	0.001	0.018	0.038	0.68
Q23 I would sacrifice for students.	**0.722**	−0.081	−0.101	0.065	0.63
Q18 I was disappointed with students not learning grammar well.	−0.009	**0.910**	0.098	0.079	0.79
Q19 When input doesn't match reward, I only do my job.	0.002	**0.785**	0.013	−0.014	0.61
Q16 I do as I like in the class I don't like.	−0.130	**0.704**	0.011	0.003	0.51
Q17 I do as I like after colleagues urge caution.	0.016	**0.638**	−0.133	−0.070	0.47
Q21 I felt lost that I couldn't persuade students.	0.094	**0.585**	0.013	0.058	0.36
Q20 I follow the leader when conflict arises.	0.042	**0.451**	−0.071	−0.011	0.23
Q03 I tried to keep calm in facing student's doubts.	0.000	−0.055	**−0.856**	−0.021	0.69
Q05 Although the leader criticized me wrongly, I didn't show it.	0.055	0.080	**−0.821**	−0.053	0.71
Q01 I tried to keep calm when encountering unknown words.	−0.009	−0.031	**−0.725**	0.010	0.52
Q04 Although I was upset with the workload, I didn't show it.	−0.044	0.137	**−0.694**	0.016	0.57
Q02 I tried to keep calm when someone listened to my class.	0.120	0.020	**−0.618**	−0.014	0.43
Q06 I didn't show my disappointment when students didn't meet my expectation.	−0.124	−0.027	**−0.608**	0.192	0.46
Q07 After consulting with master, I regulated my emotions better.	−0.088	0.070	−0.014	**0.816**	0.62
Q08 After motivating students, I was in a better state.	−0.083	0.071	−0.086	**0.804**	0.67
Q10 I tried to manage my emotions when I was upset.	0.134	−0.031	0.010	**0.757**	0.69
Q15 In the first open class, I concentrate on the class.	0.102	−0.018	0.011	**0.622**	0.46
Q11 I think from the students' standpoint when I face issues.	0.320	−0.152	−0.126	**0.447**	0.55
Q14 I do my favorite things after work.	0.283	−0.023	−0.042	**0.423**	0.42
Cumulative percentage of explained variance	30.04	46.11	54.18	58.25	—
Eigenvalues	7.284	4.101	2.245	1.307	—
Reliability of each factor	0.878	0.873	0.839	0.926	—

*The bold values indicate that the item loadings can be categorized into one factor*.

The factor accounting for the largest amount of variance (30.04%) was named “positive consonance.” It reflects the situation where teachers truly express their positive emotions, such as sincerely sacrificing for students (Q23) and openly communicating with students (Q24). In this state, teachers' internal feelings, external performance, and emotional rules are consistent. This strategy has a similar connotation with the expression of felt emotion (Ashforth and Humphrey, 1993). Six items under factor 2 reflect teachers' deliberate behavior of violating emotional rules to manage their emotions. Therefore, it is called “negative consonance.” When beginning EFL teachers encounter difficulties, they may either express their feelings truthfully or reduce emotional involvement. Specifically, they reduce their emotional involvement when they disagree with the leader (Q20) or cannot stimulate students' enthusiasm for learning English (Q21). They may show emotional deviation in their behavior when they cannot suppress their likes and dislikes toward students (Q16) or do not heed colleagues' caution (Q17). Mikolajczak et al. (2007) identified this strategy in relation to nurses' emotional labor. Moreover, Cukur's (2009) and Yang et al.'s (2019) findings supported the existence of emotional deviance and emotion termination, respectively. Factor 3 relates to teachers' management of emotions to make their behavior conform to emotional rules by disguising or suppressing negative emotions, even though their inner feelings disagree. They may use surface acting to keep calm when facing students' doubts about pronunciation (Q3) or someone suddenly visiting the classroom to listen to their class (Q2). They also use surface acting to inhibit negative emotions when students do not meet their expectations (Q6) or when they are criticized by the leader (Q5). The items under factor 4 conclude the emotion regulation skills that make their feelings conform to emotional rules, and they take the original name “deep acting” from Hochschild (1983). Using deep acting, teachers may concentrate on teaching content to reduce tension in the open class (Q15), arouse students' interest in learning to make themselves feel better (Q8), persuade themselves to adjust their emotions and not affect students (Q10), and renew cognition by thinking from the students' point of view (Q11). Moreover, the connotation of deep acting is not limited to cognitive skills, and in our study, all the techniques that make emotions concord with emotional rules were counted as deep acting. Four types of emotional labor strategies (surface acting, deep acting, positive consonance, negative consonance) were identified in this study, which slightly differs from Yin's (2012) and Li et al.'s (2012) findings, which did not include negative consonance as a strategy.

Before conducting CFA, we calculated the skewness and kurtosis values of the samples (*N* = 325) and found that the kurtosis values of items 25 and 26 were 2.605 and 2.968, respectively, far exceeding the theoretical values of between +2 and −2. Therefore, items 25 and 26 were excluded in the CFA. In addition, three items (9, 12, 13) were removed in the EFA, which were also not entered in the CFA. In other words, five items (items 9, 12, 13, 25, and 26) were excluded from the CFA. A series of CFAs was then conducted to validate the four-factor structure of the emotional labor strategies and to examine evidence of its convergent and discriminant validity. Referring to the benchmarks for indices assessing model fit (Wu, 2010b; Kline, 2016), we set the values for fit indices (χ^2^/df ≤ 8; GFI ≥ 0.90; AGFI ≥ 0.90; CFI ≥ 0.90; RMSEA ≤ 0.05; RMR ≤ 0.10). Since these indices are empirical reference values, it is necessary to determine whether the majority of fitting indices are close to or meet the basic standards. The first is the chi-square degrees of freedom ratio (χ^2^/df): the smaller the ratio, the greater the model fit. In general, a value <2 indicates a good fit of the model, but this value is affected by the sample size. Therefore, both this value and other indices should be considered when judging whether the model is acceptable (Hair et al., 2010). Since the sample size of 325 is large for CFA, 8 is appropriate for the χ^2^/df as one of the references for model adjustment.

**Figure 1:**
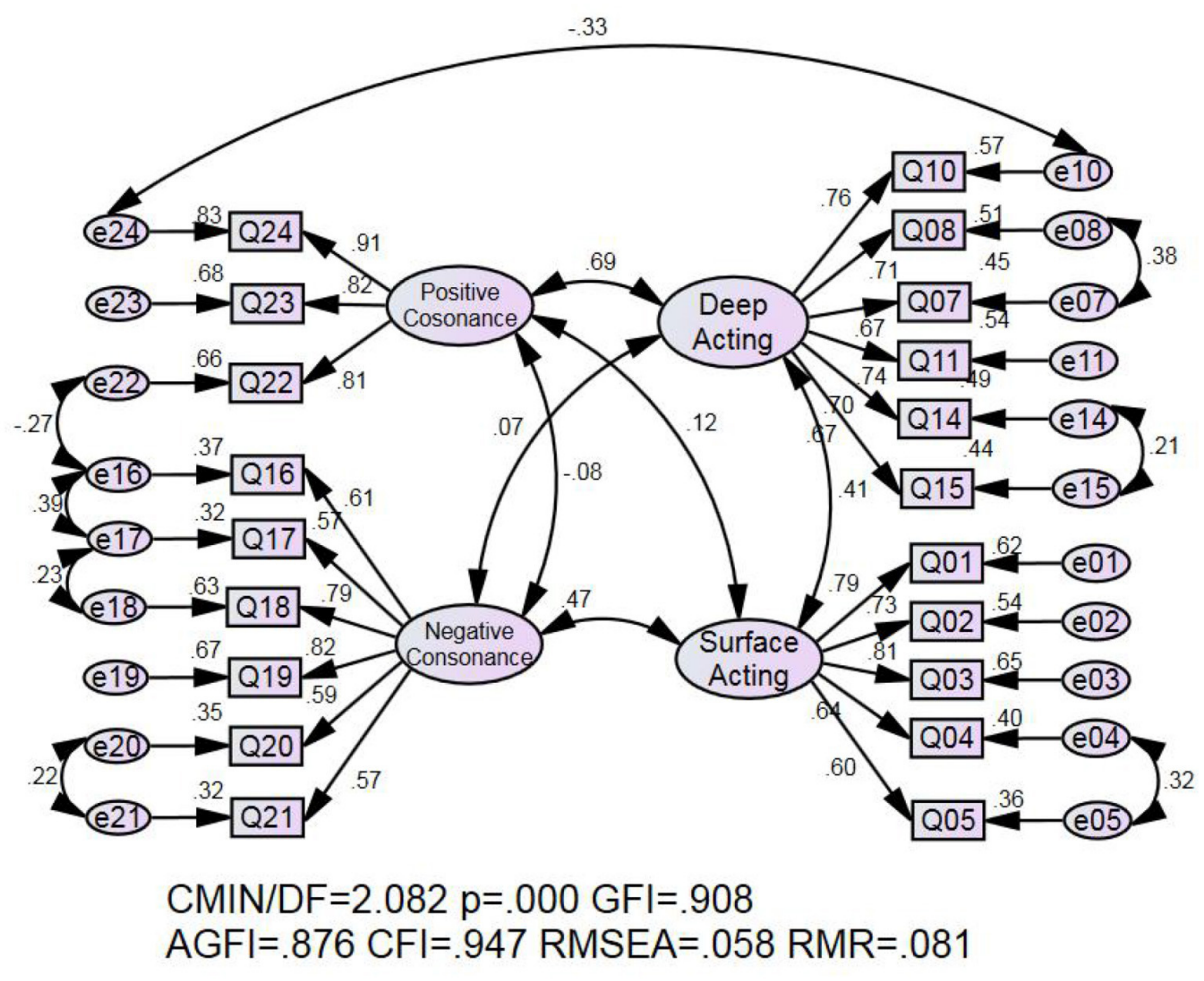
Graphical representation of the four-factor model and factor loadings.

Before conducting the CFA for the four-factor emotional labor strategy, CFAs for each factor were conducted. One item (Q6) was deleted from the surface acting dimension with loading lower than 0.40. Three rounds of CFAs for the four-factor emotional labor strategy were then performed to test whether the 20 items were in line with the four factors extracted from the EFA. The initial CFA results indicated that the 20 items and four factors were poorly supported (χ^2^/df = 2.291; GFI = 0.899; AGFI = 0.865; CFI = 0.935; RMSEA = 0.063; RMR = 0.83). The second and third rounds of CFA were conducted to adjust the covariant relationships of the models, and the results showed that the 20-item four-factor model was supported. After two rounds of model adjustment, depicted in Table 3, we compared the indices and found that the values of *p* and AGFI did not change much, but the indices of the second round were better than in the initial round and after the first adjustment. Therefore, we accept the second adjusted model as the final model of beginning EFL teachers' emotional labor strategies, composed of the four dimensions of surface acting, deep acting, positive consonance, and negative consonance (see Figure 1).

**Table 3:** CFA results of the initial analysis and two rounds of model adjustment.

**Indices**	***χ^2^*/df**	***P***	**GFI**	**AGFI**	**CFI**	**RMSEA**	**RMR**
Benchmark	≤ 8	≥0.05	≥0.90	≥0.90	≥0.90	≤ 0.08	≤ 0.10
Initial analysis	2.291	0.000	0.899	0.865	0.935	0.063	0.083
First round of adjustment	2.167	0.000	0.904	0.872	0.942	0.060	0.082
Second round of adjustment	2.082	0.000	0.908	0.876	0.947	0.058	0.081

After the CFAs, Cronbach's alpha was used to assess internal consistency for each of the sub-scales (see Appendix G). The alphas for the global scale and its four sub-scales (surface acting, deep acting, negative consonance, and positive consonance) were 0.866, 0.844, 0.865, 0.834, and 0.844, respectively. As shown in Table 4, the internal consistency values of the four dimensions were between 0.834 and 0.884, exceeding the acceptable value of 0.70 (Hair et al., 2010). The values of composite reliability were between 0.821 and 0.884, exceeding the acceptable value of 0.60 (Wu, 2010b). This means that the model has high reliability.

**Table 4:** The final distribution, composite reliability, and internal consistency of Beginning EFL Teachers' Emotional Labor Strategy Scale.

**Dimension**	**Distribution**	**Composite reliability**	**Internal consistency (Cronbach'sα)**
Surface acting (*n* = 5)	01–05	0.834	0.844
Deep acting (*n* = 6)	07–08; 10; 11; 14–15	0.857	0.865
Negative consonance (*n* = 6)	16–21	0.821	0.834
Positive consonance (*n* = 3)	22–24	0.884	0.884

For the validity of the model, we used two indicators: convergent validity and discriminant validity. Convergent validity is usually measured by the factor loading on the latent variable. It is generally believed that if the value reaches a significant level, the model can be considered to have good convergent validity (Bagozzi et al., 1991). As shown in Figure 1, the vales of each item's factor loading on the corresponding latent variable ranged from 0.57 to 0.91, and all reached the significance level of 0.001 (see Appendix H for details). We adopted the average variance extracted (AVE) method to test the discriminant validity between sub-scales. Specifically, we compared the square root of the AVE of one sub-scale with its correlation coefficient (Wu, 2010b; Kline, 2016). We examined the correlations among the four sub-scales, as shown in Table 5. Surface acting correlated positively with deep acting (γ = 0.390, *p* < 0.01) and positively with negative consonance (γ = 0.428, *p* < 0.01). Although surface acting correlated positively with positive consonance (γ = 0.106, *ns*), it was not significant. Deep acting correlated positively with negative consonance (γ = 0.114, *p* < 0.05) and positive consonance (γ = 0.581, *p* < 0.01). Negative consonance correlated negatively with positive consonance non-significantly (γ = −0.06, *ns*). The square roots of the AVE for surface acting, deep acting, negative consonance, and positive consonance were 0.725, 0.720, 0.683, and 0.084, respectively. Since the values of the square root of the AVE for every sub-scale were larger than all the correlation coefficients among the sub-scales, all dimensions possessed good discriminant validity (Brown, 2006).

**Table 5:** Discriminant validity of each sub-scale of the Beginning EFL Teachers' Emotional Labor Strategy Scale.

**Sub-scale**	**1**	**2**	**3**	**4**	**Square root of the AVE**
Surface acting	—				0.725
Deep acting	0.390^**^	—			0.720
Negative consonance	0.428^**^	0.114^*^	—		0.683
Positive consonance	0.106	0.581^**^	−0.06	—	0.849

** *P < 0.01*;

* *P < 0.05*.

The CFA results further verified the four-factor structure of beginning EFL teachers' emotional labor strategies naturally extracted by EFA. More specifically, the beginning EFL teachers' emotional labor strategy is a multidimensional construct that reflects various processes of emotion regulation, including surface acting, deep acting, negative consonance, and positive consonance. Surface acting refers to a strategy in which teachers pretend to feel emotions that they do not actually feel or inhibit negative emotions. Deep acting refers to both cognitive and behavioral emotion regulation skills that make teachers' feelings conform to emotional rules. Negative consonance refers to the emotion regulation methods of emotional deviance and emotion termination, by which teachers ignore emotional rules to regulate emotions. Positive consonance refers to the situation where teachers truly express their positive emotions, and the teachers' internal feelings, external performance, and emotional rules are consistent. A series of tests confirmed the high reliability and validity of the model.

### Levels of Emotional Labor Strategies

To answer the second research question—“What are the overall and dimensional profiles of their emotional labor strategies?”—we conducted descriptive statistical analyses and calculated the mean scores of the Beginning EFL Teachers' Emotional Labor Strategy Scale and its sub-scales. As presented in Table 6, the results of the whole scale indicated moderate use of emotional labor strategies (*M* = 67.112, *SD* = 11.722). Teachers were most favorable to using positive consonance (*M* = 4.151, *SD* = 0.832), then to deep acting (*M* = 3.923, *SD* = 0.774), followed by surface acting (*M* = 2.998, *SD* = 0.995), and they were least favorable to negative consonance (*M* = 2.689, *SD* = 0.840). The values of both surface acting and negative consonance were below the median of 3.

**Table 6:** Descriptive statistics of the Beginning EFL Teachers' Emotional Labor Strategy Scale (*N* = 325).

**Sub-scale**	***M***	***SD***	***Min***	***Max***
Surface acting	2.998	0.995	1.000	5.000
Deep acting	3.923	0.774	1.000	5.000
Negative consonance	2.689	0.840	1.000	5.000
Positive consonance	4.151	0.832	1.000	5.000
Whole scale	67.112	11.722	20.000	100.000

Generally speaking, beginning EFL teachers in Chinese secondary schools make more use of positive consonance and deep acting to regulate emotions, which implies that in most circumstances, beginning teachers' feelings and behaviors at work are consistent with the emotional rules. Applying these two types of strategies has positive significance (Zhang and Zhu, 2008; Wang et al., 2019), reflecting their efforts to adapt to the work. Comparatively speaking, the use of negative consonance and surface acting was infrequent. The above status reflects that the beginning EFL teachers take teaching seriously, sincerely care for students, and actively coordinate interpersonal relationships in their work.

## Discussion

This study validates, for the first time, the dimensions of beginning EFL teachers' emotional labor strategies. Hence, it responds to researchers' (e.g., Cukur, 2009; Yin, 2012; Wang et al., 2019) call for developing measuring instruments and provides an operational conceptualization of beginning EFL teachers' emotional labor strategies. After developing the Beginning EFL Teachers' Emotional Labor Strategy Scale, series of EFAs and CFAs clarified that the emotional labor strategy has a four-factor structure. It consists of surface acting, deep acting, negative consonance, and positive consonance. This conclusion is consistent with Mikolajczak et al.'s (2007) four-dimension emotional labor strategy and partially confirms Cukur's (2009) and Yang et al.'s (2019) points that the violation of emotional rules should be considered as ways to manage emotions. This structural division is in line with the objective reality of beginning EFL teachers in Chinese secondary schools, which is helpful for understanding the different psychological processes underpinning their emotional labor. Although they have aspirations for education, they are more likely to feel negative emotions than experienced teachers because of the conflict of role conversion, lack of teaching experience, and professional competence. Therefore, when they encounter problems beyond their ability, some beginning teachers may adopt negative consonance to manage their emotions. Essentially, this strategy reflects the helplessness of beginning teachers and reveals the difficulties they encounter at work, such as managing students, having no clues about how to improve students' learning effects, and not knowing how to get along with leaders. This is consistent with the findings of previous qualitative studies on beginning EFL teachers (Xu, 2013; Tejeda et al., 2016).

Compared with extant teachers' emotional labor strategy scales (e.g., Cukur, 2009; Li et al., 2012; Yin, 2012), the Beginning EFL Teachers' Emotional Labor Strategy Scale is closer to the teaching realities of Chinese beginning EFL teachers in secondary schools, mirroring their interpersonal communication processes. For example, they may encounter situations where students doubt their pronunciation, where they feel nervous about unknown English words while teaching, and where they panic in their first open class. At the same time, the scale reflects the reality that they are deeply influenced by traditional Chinese Confucianism although they teach English. China has always held a common belief that teachers not only bear the responsibilities of preaching, teaching, and dispelling doubts but also the conviction that “a teacher for a day is a father for life.” Therefore, they have absolute authority and high social status. Different from Western cultures, it is acceptable in the Chinese culture for teachers to express negative emotions to students (Yin and Lee, 2012). One dimension of the scale, “negative consonance,” reflects this behavior. The beginning EFL teachers may choose to violate emotional rules to manage their emotions or achieve teaching purposes (Yin, 2016), causing them to express negative emotions or emotions that defy expectations. The use of this strategy is also consistent with some existing research on Chinese teachers (e.g., Yin, 2016; Yin et al., 2017; Liang, 2019). Chinese teachers usually are strict with students while caring for them. Therefore, they will use these four strategies selectively: surface acting to maintain distance between themselves and students, negative consonance to maintain their authority and command respect from students, and deep acting and positive consonance to show care and love.

The teachers' scores on the four emotional labor strategies show that they are more inclined to use positive consonance and deep acting to regulate their emotions, less favorable to using surface acting, and least favorable to using negative consonance. Cukur (2009) also found that teachers use strategies that deviate from emotional rules the least frequently. This indicates that beginning EFL teachers can adjust their emotions according to the emotional rules in most cases and will seldom use strategies deviating from emotional rules to adjust their emotions. In terms of the other three strategies that comply with emotional rules, both Yin et al.'s (2017) and Zhang and Zhu's (2008) studies found that Chinese teachers used surface acting the least often. This may be because teachers and students in China spend a long time together, making it difficult for a teacher to fake or suppress emotions during these long-term, high-frequency interactions. Yin et al. (2017) took 115 primary school English teachers in Hong Kong as their research subjects, while Zhang and Zhu (2008) chose Chinese college instructors as participants. The current research is in accord with their findings that teachers engage more in deep acting than surface acting. This highlights the efficiency of deep acting for emotion management, no matter what teaching backgrounds teachers may have. By engaging in deep acting, most teachers could realize the conformity of their feelings and expressions. The positive consonance in this study has a similar meaning with a genuine expression of emotions; that is, the emotions felt and expressed are consistent with the emotional rules. Both the participants in this study and the primary English teachers in Yin et al.'s (2017) research were inclined to express their true feelings using the strategy of genuine expression of emotions or, in this case, positive consonance. The reason may be that primary and secondary school teachers spend more time with students; therefore, they have more space to express their true feelings. In addition, their teaching targets are less mature and need more care. Therefore, it is necessary for teachers to express their positive attitudes more directly to enable students to feel their love. In contrast, Zhang and Zhu's (2008) study found that college EFL teachers used more deep acting than genuine expression of emotions. This may be because college students are basically adults, and college teachers need to pay attention to concerns about face and harmonious teacher–student relationships. In addition, since the participants in the present study were beginning teachers, they were young and eager to be recognized as good teachers and helpful friends to students (Xu, 2013). To some extent, this starting point and the mode of getting along with students affect the types of emotional labor strategies teachers apply. In the process of getting along with students, they may use more mitigation strategies, such as positive consonance and deep acting, to narrow the distance with students.

## Conclusion and Implications

This study verified the four dimensions of emotional labor strategies through factor analyses and constructed a four-dimensional model of beginning EFL teachers' emotional labor strategy comprising surface acting, deep acting, positive consonance, and negative consonance. The model has high reliability and discriminant validity. Using the data of the final model, we conducted descriptive statistical analyses to analyze the current situation of Chinese beginning EFL teachers' use of emotional labor strategies. The results show that these teachers use positive consonance the most, followed by deep acting, surface acting, and negative consonance. This shows that most of the beginning EFL teachers in secondary schools can actively regulate their emotions according to emotional rules and only a few of them violate the emotional rules. On the whole, the professional quality of beginning EFL teachers in Chinese secondary schools is relatively high, and they can actively perform emotional labor.

The results of this study have several practical implications in terms of enhancing beginning EFL teachers' ability to manage their emotions. For the above purpose, we also put forward some suggestions in the hope of helping them better undertake emotional labor and achieve their own sustainable development (Derakhshan and Shirmohammadli, 2015; Chu et al., 2021). First, the Beginning EFL Teachers' Emotional Labor Strategy Scale developed here is a concise multiple-item scale with good reliability and validity, which is based on the realities that Chinese beginning secondary school EFL teachers face. China's related organizations could use this scale to better understand the emotional labor that teachers perform and take actions to improve their emotional capacity. As for secondary schools, they should provide a comfortable working environment for teachers, arrange work tasks reasonably, and create favorable conditions for the development of beginning teachers. Specifically, schools should consider teachers' long-term development. They can help them improve their teaching abilities first and then let them participate in school construction. Schools should also establish a practice community to help teachers grow, for example, by assigning masters, providing psychological counseling, and coordinating communication with parents and students. This can reduce teachers' burden of daily work and enhance parents' and students' understanding. In addition, teacher education programs could assist teachers by equipping them with emotion regulation skills. Only in this way can beginning teachers thrive in their professional lives.

Second, the scale lists examples of difficulties beginning EFL teachers may encounter at work and the corresponding strategies to deal with such situations. Teachers could use this scale to test their ability to do emotional labor, thereby knowing themselves better. It is necessary for them to learn the types and differences of these emotional labor strategies. Unpacking their choices of different strategies could provide useful insights into emotion regulation (Greenier et al., 2021). Beginning EFL teachers should improve their teaching and emotion regulation abilities. Only when beginning secondary school EFL teachers improve their abilities of regulating emotions, interpersonal communication, and teaching can they solve problems fundamentally.

Despite the above implications and suggestions, this research has some limitations. First, data collected from both interviews and scales were self-reported, which can result in consistency motif or social desirability (Podsakoff and Organ, 1986). The participants may have had an urge to maintain consistent answers or present themselves in a favorable way. Future research could enrich the sources of data by adopting observation or journal entries. Second, although a series of tests was conducted to confirm the scale's reliability and validity, replication with different samples is necessary to further support this measurement of emotional labor strategies for Chinese secondary school beginning EFL teachers. Third, only 39 male (8.1%) teachers participated in the survey, which shows an unbalanced gender distribution. Although it may actually reflect the status of gender distribution among Chinese beginning secondary school EFL teachers, the potential influence of gender ratios must still be tested in future studies.

## Data Availability Statement

The original contributions presented in the study are included in the article/Supplementary Files, further inquiries can be directed to the corresponding author/s.

## Ethics Statement

The studies involving human participants were reviewed and approved by School of Foreign Languages, Northeast Normal University, China. The patients/participants provided their written informed consent to participate in this study. Written informed consent was obtained from the individual(s) for the publication of any potentially identifiable images or data included in this article.

## Author Contributions

HLi and HLiu contributed to the research and paper writing. Both authors contributed to the article and approved the submitted version.

## Conflict of Interest

The authors declare that the research was conducted in the absence of any commercial or financial relationships that could be construed as a potential conflict of interest.

## Publisher's Note

All claims expressed in this article are solely those of the authors and do not necessarily represent those of their affiliated organizations, or those of the publisher, the editors and the reviewers. Any product that may be evaluated in this article, or claim that may be made by its manufacturer, is not guaranteed or endorsed by the publisher.
