# Supplementary material for: Beginning EFL Teachers' Emotional Labor Strategies in the Chinese Context
Source: Front Psychol. 2021 Aug 17;12:737746. doi: 10.3389/fpsyg.2021.737746 (PMC8418064; doi:10.3389/fpsyg.2021.737746)
Supplement: Supplementary file 1 [file Table_1.doc]

**Appendix A Beginning English Teachers’ Emotional labour Strategies Scale**

| Q01 | Although I was nervous when I met English words I didn’t know in class, I still tried my best to keep calm. |
| --- | --- |
| Q02 | I tried my best to stay calm even though I was nervous when someone randomly listened to my class (such as leaders and colleagues). |
| Q03 | Although embarrassed by the student’s doubts about my pronunciation, I tried to appear calm . |
| Q04 | I was assigned a lot of tasks unrelated to teaching. Although I felt helpless, I tried not to show it. |
| Q05 | When leader criticized me, although I was wronged, I tried not to show it. |
| Q07 | After learning from my master, I was able to adjust my emotions better. |
| Q08 | After evoking students’ learning initiative, the quality of my teaching has also changed for the better. |
| Q10 | When I am in a bad mood, I will tell myself that I should try my best to adjust my emotions and try not to affect students. |
| Q11 | When I encounter problems, I will try to think from students’ standpoint. |
| Q14 | When I encounter unpleasant things at work, I will do what I like to do after work to adjust my emotions. |
| Q15 | In the first open class, I tried to concentrate on the class and make myself not so nervous. |
| Q16 | When I’m in a class I don’t like, I tend to act on my feelings. |
| Q17 | After my colleagues reminded me to pay attention to the way I deal with students, I am more inclined to act according to my own feelings. |
| Q18 | I tend to show my disappointment when students don’t master the grammatical points that have been taught many times. |
| Q19 | When I feel that the give is not proportional to the take, I choose to complete my own work. |
| Q20 | When disagreement occurs, I choose to follow leader’s requirements and not to express my emotions. |
| Q21 | After many times of persuading the students who do not attach importance to English learning, I feel at a loss. |
| Q22 | My love for students comes from my heart. |
| Q23 | No matter how much I invest in students, I am willing to do it. |
| Q24 | I can completely open my heart to communicate with students and establish a harmonious relationship with students. |

**Appendix B The refined coding system of emotional labour strategies**

| Strategies | Sub-strategies | Examples of Strategies |
| --- | --- | --- |
| surface acting | pretending | Although I was nervous when I met English words I didn’t know in class, I still tried my best to keep calm. |
|  | inhibition | I was assigned a lot of tasks unrelated to teaching. Although I felt helpless, I tried not to show it. |
| deep acting | taking action | After learning from my master, I was able to adjust my emotions better. |
|  | cognitive reconstruction | If I encounter problems, I will try to think from the standpoint of students. |
|  | emotional persuasion | When I am in a bad mood, I will tell myself that I should try my best to adjust my mood and not to affect the students. |
|  | emotional dissociation | When I encounter unpleasant things at work, I will do what I like to do after work to adjust my mood. |
| positive consonance | releasing positive emotions | The emotions I show students match what I spontaneously feel. |
| negative consonance | emotional deviation | I tend to show my disappointment when students don’t master the English grammar points that have been taught many times. |
|  | emotional termination | Although I disagree with leader, I choose to follow his/her requirement, showing no emotions. |

**Appendix C Beginning English Teachers’ Emotional labour Strategies Scale for EFA**

| Q01 | Although I was nervous when I encountered English words I didn’t know in class, I still tried my best to keep calm. |
| --- | --- |
| Q02 | I tried my best to stay calm even though I was nervous when someone randomly listened to my class (such as leaders and colleagues). |
| Q03 | Although I was embarrassed by the student’s doubts about my pronunciation, I tried to appear calm. |
| Q04 | I was assigned a lot of tasks unrelated to teaching. Although I felt helpless, I tried not to show it. |
| Q05 | When the leader criticized me, although I felt wronged, I tried not to show it. |
| Q06 | When students do not meet my expectations, although I am disappointed, I try not to show it. |
| Q07 | After learning from my master, I was able to adjust my emotions better. |
| Q08 | After evoking students’ learning initiative, the quality of my teaching has also changed for the better. |
| Q09 | When I can’t answer students’ questions, I will tell myself that beginning teachers will encounter similar problems, which is nothing. |
| Q10 | When I am in a bad mood, I will tell myself that I should try my best to adjust my emotions and try not to affect students. |
| Q11 | When I encounter problems, I will try to think from the students’ standpoint. |
| Q12 | When students’ grades are not ideal, I will find some objective causes to make myself feel better. |
| Q13 | Even if something bad happened to my family, I still tried to focus on my work. |
| Q14 | When I encounter unpleasant things at work, I will do what I like to do after work to adjust my emotions. |
| Q15 | In the first open class, I tried to concentrate on the class and make myself not so nervous. |
| Q16 | When I’m in a class I don’t like, I tend to act on my feelings. |
| Q17 | After my colleagues reminded me to pay attention to the way I deal with students, I am more inclined to act according to my own feelings. |
| Q18 | I tend to show my disappointment when students don’t master the grammatical points that have been taught many times. |
| Q19 | When I feel that the give is not proportional to the take, I choose to complete my own work. |
| Q20 | When disagreement occurs, I choose to follow the leader’s requirements and not to express my emotions. |
| Q21 | After many attempts to persuade the students who do not attach importance to English learning, I feel at a loss. |
| Q22 | My love for students comes from my heart. |
| Q23 | No matter how much I invest in students, I am willing to do it. |
| Q24 | I can completely open my heart to communicate with students and establish a harmonious relationship with them. |
| Q25 | When students have a positive response to class, my enthusiasm for teaching is even higher. |
| Q26 | If students’ English level has improved, I will be more confident. |

**Appendix D Critical ration,correlation matrix of each item and the global scale**

| Item | CR | Global | Item | CR | Global |
| --- | --- | --- | --- | --- | --- |
| 1 | -8.267^**^ | 0.594^**^ | 14 | -6.686^**^ | 0.499^**^ |
| 2 | -9.707^**^ | 0.615^**^ | 15 | -6.403^**^ | 0.503^**^ |
| 3 | -12.560^**^ | 0.663^**^ | 16 | -3.224^**^ | 0.306^**^ |
| 4 | -9.917^**^ | 0.646^**^ | 17 | -4.718^**^ | 0.398^**^ |
| 5 | -13.619^**^ | 0.748^**^ | 18 | -4.891^**^ | 0.427^**^ |
| 6 | -7.102^**^ | 0.553^**^ | 19 | -4.068^**^ | 0.350^**^ |
| 7 | -7.915^**^ | 0.596^**^ | 20 | -3.460^**^ | 0.306^**^ |
| 8 | -8.486^**^ | 0.607^**^ | 21 | -5.460^**^ | 0.427^**^ |
| 9 | -7.106^**^ | 0.551^**^ | 22 | -5.570^**^ | 0.429^**^ |
| 10 | -7.835^**^ | 0.570^**^ | 23 | -6.394^**^ | 0.466^**^ |
| 11  12  13 | -6.931^**^ | 0.469^**^ | 24 | -6.149^**^ | 0.411^**^ |
|  | -6.620^**^ | 0.486^**^ | 25 | -5.135^**^ | 0.424^**^ |
|  | -6.570^**^ | 0.502^**^ | 26 | -5.194^**^ | 0.454^**^ |

**p*<0.05, ***p*<0.01, ****p*<0.001

**Appendix E Normal distribution test of data before Exploratory Factor Analysis**

| **Item** | Skewness | | Kurtosis | |
| --- | --- | --- | --- | --- |
|  | Statistic | Std. Error | Statistic | Std. Error |
| Q01 | .090 | .192 | -.973 | .383 |
| Q02 | -.445 | .192 | -.642 | .383 |
| Q03 | .057 | .192 | -1.235 | .383 |
| Q04 | -.202 | .192 | -.918 | .383 |
| Q05 | -.146 | .192 | -1.125 | .383 |
| Q06 | -.010 | .192 | -.804 | .383 |
| Q07 | -.992 | .192 | .790 | .383 |
| Q08 | -1.192 | .192 | 1.470 | .383 |
| Q09 | .066 | .192 | -1.105 | .383 |
| Q10 | -.781 | .192 | .110 | .383 |
| Q11 | -.541 | .192 | -.137 | .383 |
| Q12 | .090 | .192 | -.690 | .383 |
| Q13 | -.391 | .192 | -.666 | .383 |
| Q14 | -.465 | .192 | -.407 | .383 |
| Q15 | -.642 | .192 | .373 | .383 |
| Q16 | .533 | .192 | -.517 | .383 |
| Q17 | .491 | .192 | -.366 | .383 |
| Q18 | .205 | .192 | -.473 | .383 |
| Q19 | .270 | .192 | -.662 | .383 |
| Q20 | -.094 | .192 | -.261 | .383 |
| Q21 | -.227 | .192 | -.410 | .383 |
| Q22 | -1.089 | .192 | .449 | .383 |
| Q23 | -.533 | .192 | -.751 | .383 |
| Q24 | -.831 | .192 | .168 | .383 |
| Q25 | -1.458 | .192 | 1.797 | .383 |
| Q26 | -1.533 | .192 | 1.933 | .383 |

**Appendix F** Reliability values of each dimension after Exploratory Factor Analysis

Surface Acting

|  |  | Scale Mean if Item Deleted | Scale Variance if Item Deleted | Corrected Item-Total Correlation | Cronbach's Alpha if Item Deleted |
| --- | --- | --- | --- | --- | --- |
| Q01 | Nervous about unknown words | 16.03 | 25.043 | .670 | .859 |
| Q02 | Nervous when someone listened to class | 15.62 | 26.376 | .603 | .870 |
| Q03 | Embarrassed by students’ doubt | 16.14 | 23.069 | .762 | .843 |
| Q04 | Helpless about tasks | 15.92 | 24.595 | .693 | .855 |
| Q05 | Show no emotions when criticized wrongly | 16.02 | 23.107 | .771 | .841 |
| Q06 | Show no emotions when students fail | 15.93 | 26.723 | .606 | .869 |

Deep Acting

|  |  | Scale Mean if Item Deleted | Scale Variance if Item Deleted | Corrected Item-Total Correlation | Cronbach's Alpha if Item Deleted |
| --- | --- | --- | --- | --- | --- |
| Q07 | Learn from master | 19.62 | 14.263 | .676 | .851 |
| Q08 | Evoke students | 19.38 | 14.567 | .707 | .845 |
| Q10 | Self persuade | 19.61 | 13.531 | .774 | .832 |
| Q11 | Think from students’ points | 19.57 | 15.310 | .642 | .856 |
| Q14 | Do things after work | 19.59 | 15.408 | .596 | .864 |
| Q15 | Concentrate on class at first open class | 19.65 | 14.911 | .652 | .855 |

Negative Consonance

|  |  | Scale Mean if Item Deleted | Scale Variance if Item Deleted | Corrected Item-Total Correlation | Cronbach's Alpha if Item Deleted |
| --- | --- | --- | --- | --- | --- |
| Q16 | Act on my feelings at my less favourable class | 14.72 | 17.116 | .640 | .807 |
| Q17 | Act on my feelings to deal with students | 14.59 | 17.560 | .628 | .810 |
| Q18 | show my disappointment for students’ performance | 14.43 | 16.500 | .794 | .776 |
| Q19 | Finish own task | 14.42 | 16.663 | .704 | .793 |
| Q20 | follow leader’s requirements | 13.92 | 19.918 | .423 | .846 |
| Q21 | feel at a loss for not persuading students | 13.86 | 18.530 | .509 | .833 |

Positive Consonance

|  |  | Scale Mean if Item Deleted | Scale Variance if Item Deleted | Corrected Item-Total Correlation | Cronbach's Alpha if Item Deleted |
| --- | --- | --- | --- | --- | --- |
| Q22 | Love students sincerely | 17.14 | 8.740 | .828 | .905 |
| Q23 | Willing to help students | 17.48 | 8.314 | .770 | .920 |
| Q24 | Open heart to communicate | 17.35 | 8.405 | .808 | .910 |
| Q25 | Enthusiastic about students response | 17.00 | 9.127 | .833 | .906 |
| Q26 | Confident about students’ | 17.00 | 9.013 | .827 | .907 |

**Appendix G** Reliability values of each dimension after Confirmatory Factor Analysis

Surface Acting

|  |  | Scale Mean if Item Deleted | Scale Variance if Item Deleted | Corrected Item-Total Correlation | Cronbach's Alpha if Item Deleted |
| --- | --- | --- | --- | --- | --- |
| Q01 | Nervous about unknown words | 12.10 | 16.624 | .666 | .809 |
| Q02 | Nervous when someone listened to class | 11.66 | 17.022 | .655 | .812 |
| Q03 | Embarrassed by students’ doubt | 12.36 | 15.564 | .710 | .796 |
| Q04 | Helpless about tasks | 11.87 | 16.263 | .638 | .817 |
| Q05 | Show no emotions when criticized wrongly | 11.98 | 16.849 | .591 | .829 |

Deep Acting

|  |  | Scale Mean if Item Deleted | Scale Variance if Item Deleted | Corrected Item-Total Correlation | Cronbach's Alpha if Item Deleted |
| --- | --- | --- | --- | --- | --- |
| Q07 | Learn from master | 19.68 | 15.337 | .645 | .845 |
| Q08 | Evoke students | 19.50 | 15.399 | .695 | .836 |
| Q10 | Self persuade | 19.70 | 14.698 | .678 | .839 |
| Q11 | Think from students’ points | 19.54 | 15.879 | .663 | .842 |
| Q14 | Do things after work | 19.61 | 15.418 | .668 | .841 |
| Q15 | Concentrate on class at first open class | 19.67 | 15.469 | .618 | .850 |

Negative Consonance

|  |  | Scale Mean if Item Deleted | Scale Variance if Item Deleted | Corrected Item-Total Correlation | Cronbach's Alpha if Item Deleted |
| --- | --- | --- | --- | --- | --- |
| Q16 | Act on my feelings at my less favourable class | 13.70 | 17.888 | .581 | .814 |
| Q17 | Act on my feelings to deal with students | 13.76 | 18.243 | .605 | .808 |
| Q18 | show my disappointment for students’ performance | 13.52 | 17.849 | .705 | .788 |
| Q19 | Finish own task | 13.53 | 17.225 | .703 | .787 |
| Q20 | follow leader’s requirements | 13.09 | 19.273 | .533 | .822 |
| Q21 | feel at a loss for not persuading students | 13.06 | 18.950 | .528 | .823 |

Positive Consonance

|  |  | Scale Mean if Item Deleted | Scale Variance if Item Deleted | Corrected Item-Total Correlation | Cronbach's Alpha if Item Deleted |
| --- | --- | --- | --- | --- | --- |
| Q22 | Love students sincerely | 8.15 | 3.091 | .750 | .856 |
| Q23 | Willing to help students | 8.44 | 2.797 | .761 | .850 |
| Q24 | Open heart to communicate | 8.31 | 2.901 | .816 | .799 |

**Appendix H Factor loading of each variable after Confirmatory Factor Analysis**

|  | Estimate | S.E. | C.R. | P | Label |
| --- | --- | --- | --- | --- | --- |
| Positive consonance | .522 | .060 | 8.750 | *** |  |
| Negative consonance | .917 | .112 | 8.199 | *** |  |
| Surface acting | .929 | .117 | 7.961 | *** |  |
| Deep acting | .466 | .067 | 6.975 | *** |  |
| e22 | .271 | .027 | 9.980 | *** |  |
| e23 | .306 | .031 | 9.751 | *** |  |
| e24 | .139 | .023 | 5.935 | *** |  |
| e19 | .449 | .059 | 7.618 | *** |  |
| e18 | .429 | .051 | 8.429 | *** |  |
| e17 | .880 | .077 | 11.434 | *** |  |
| e01 | .561 | .062 | 9.106 | *** |  |
| e02 | .636 | .062 | 10.199 | *** |  |
| e03 | .614 | .071 | 8.652 | *** |  |
| e08 | .449 | .042 | 10.635 | *** |  |
| e07 | .574 | .052 | 11.029 | *** |  |
| e10 | .506 | .051 | 9.929 | *** |  |
| e20 | .740 | .065 | 11.359 | *** |  |
| e11 | .381 | .037 | 10.439 | *** |  |
| e14 | .491 | .046 | 10.726 | *** |  |
| e04 | 1.028 | .092 | 11.210 | *** |  |
| e16 | .933 | .082 | 11.418 | *** |  |
| e21 | .864 | .075 | 11.517 | *** |  |
| e15 | .590 | .053 | 11.034 | *** |  |
| e05 | 1.059 | .093 | 11.449 | *** |  |
